# Secreted *Mycobacterium tuberculosis* Rv3654c and Rv3655c Proteins Participate in the Suppression of Macrophage Apoptosis

**DOI:** 10.1371/journal.pone.0010474

**Published:** 2010-05-04

**Authors:** Lia Danelishvili, Yoshitaka Yamazaki, Jeannie Selker, Luiz E. Bermudez

**Affiliations:** 1 Department of Biomedical Sciences, College of Veterinary Medicine, Oregon State University, Corvallis, Oregon, United States of America; 2 Department of Infectious Diseases and Laboratory Medicine, School of Medicine, Shinshu University, Matsumoto, Japan; 3 Institute of Molecular Biology, University of Oregon, Eugene, Oregon, United States of America; 4 Department of Microbiology, College of Science, Oregon State University, Corvallis, Oregon, United States of America; National Institute for Infectious Diseases L. Spallanzani, Italy

## Abstract

**Background:**

Inhibition of macrophage apoptosis by *Mycobacterium tuberculosis* has been proposed as one of the virulence mechanisms whereby the pathogen avoids the host defense. The mechanisms by which *M. tuberculosis* H37Rv strain suppress apoptosis and escapes human macrophage killing was investigated.

**Methodology/Principal Findings:**

The screening of a transposon mutant bank identified several mutants, which, in contrast to the wild-type bacterium, had impaired ability to inhibit apoptosis of macrophages. Among the identified genes, *Rv3659c* (31G12 mutant) belongs to an operon reminiscent of type IV pili. The Rv3654c and Rv3655c putative proteins in a seven-gene operon are secreted into the macrophage cytoplasm and suppress apoptosis by blocking the extrinsic pathway. The operon is highly expressed when the bacterium is within macrophages, compared to the expression level in the extracellular environment. Rv3654c recognizes the polypyrimidine tract binding Protein-associated Splicing Factor (PSF) and cleaves it, diminishing the availability of caspase-8. While *M. tuberculosis* inhibits apoptosis by the extrinsic pathway, the pathogen does not appear to affect the intrinsic pathway. Inactivation of the intrinsic pathway by pharmacologic agents afftects *M. tuberculosis* and induces cell necrosis. Likewise, inactivation of PSF by siRNA significantly decreased the level of caspase-8 in macrophages.

**Conclusion:**

While *M. tuberculosis* inhibits the extrinsic pathway of apoptosis, it appears to activate the intrinsic pathway leading to macrophage necrosis as a potential exit strategy.

## Introduction

*Mycobacterium tuberculosis* is an intracellular pathogen infecting primarily mononuclear phagocytes. The bacterium has developed intricate strategies to evade killing mechanisms of phagocytes [1]. Once *M. tuberculosis* is inhaled and deposited in the alveolar space, it infects alveolar macrophages, prevents phagosome acidification and subsequent fusion of the vacuole with lysosomes [2], [3]. Macrophages control bacterial growth by a number of mechanisms, including the production of reactive oxygen intermediaries and nitric oxide (NO) [4]. *M. tuberculosis* blocks the delivery of nitric oxide synthase (iNOS) to the vacuole membrane and consequently avoids the toxic effects of NO [5]; although NO still has some impact on the bacterial viability, as evidenced by the increase in bacterial survival in iNOS knockout mice [5]. Autophagy, a repair mechanism of eukaryotic cells, frequently associated with cell starvation, has also recently been shown to eliminate intracellular bacteria through the delivery of ubiquitin-derived peptide to the mycobacterial vacuoles [6], [7]. A number of reviews have explored the role of autophagy in host defense against infectious agents [8]. Autophagy is connected through several possible pathways with apoptosis [8]. It is plausible to assume that, when infected macrophages fail to kill *M. tuberculosis* by autophagy, the alternative host strategy, apoptosis, is used to eliminate the intracellular bacteria [9]. *M. tuberculosis* has the ability to suppress macrophage apoptosis using active mechanisms, with the aim of blocking the expression of apoptosis-associated genes or stimulating anti-apoptosis pathways [10], [11]. The importance of apoptosis as a host-defense strategy has also been studied *in vivo* [12]. In one of these studies, while macrophages isolated from mice with resistant *sst1* (super-susceptibility to tuberculosis-1) locus undergo apoptosis in response to *M. tuberculosis* infection, macrophages of *sst1*-susceptible mice die after infection and show widespread necrosis [12]. A recent study suggested that both H37Rv and H37Ra strains of *M. tuberculosis* disrupt the mitochondrial outer membrane, but only the virulent H37Rv induces significant mitochondrial transmembrane potential loss [13]. Other studies have concentrated their observations on focused aspects of the pathways [9], [11], but none thus far have used gene knockout mutants to dissect the possible mechanisms involved. Only recently, a report by Velmurugan and colleagues attempted to investigate a H37Rv mutant clone deficient in suppression of apoptosis [14].

The underlying mechanisms by which virulent *M. tuberculosis* inhibits apoptosis in macrophages remain largely unknown. Macrophages undergo apoptosis by at least two different pathways. The extrinsic pathway, in which released tumor necrosis factor-alpha (TNF-α), activates caspase-8. The adaptor proteins TRADD, RIP-1 or FAAD (TRADD in the case of TNF-α stimulus), stimulate the downstream caspases, through the type I (extrinsic) or type II (intrinsic) pathways [15], [16], [17]. Recent work by Kundu and colleagues showed that *M. tuberculosis*-induced apoptosis is mediated by p38 and ASK-1 (apoptosis signal-regulating kinase 1), and that the pathogen also induces degradation of FLiPS, which, when ubiquitinated, blocks TNF-α-mediated apoptosis [18]. In contrast, Chen and colleagues described that the virulent H37Rv strain stimulates LxA4, which inhibits PGE_2_ production . PGE_2_ protects mitochondrial membranes from damage and inhibits necrosis [19].

The discordant findings in the literature, regarding *M. tuberculosis* inducing or inhibiting apoptosis, may be related to a large variety of factors, such as cell types, strain phenotype and phases of infection when the studies are carried out. Because of the inconsistency, we decided to investigate the ability and mechanism by which virulent *M. tuberculosis* H37Rv strain suppresses apoptosis and escapes killing by human macrophages, by using the screening of a transposon library to identify mutant(s) that do not inhibited the apoptosis. Previous studies establish that inhibition of apoptosis by *M. tuberculosis* is seen in U937, THP-1 and human monocyte-cleaved macrophages [10]. In the present study, we demonstrate that *M. tuberculosis* is capable of blocking the extrinsic pathway of apoptosis by secreting effector proteins that interfere with caspases' post-transcriptional events.

## Materials and Methods

### Bacterial strain, cell culture and infections

Mouse-passed, virulent *Mycobacterium tuberculosis* strain H37Rv (ATCC 25618) was cultured for 3 weeks in liquid Middlebrook 7H9 medium or Middlebrook 7H10 agar plates supplemented with 10% oleic acid, albumin, dextrose and catalase (OADC) enrichment (Hardy Diagnostics, Santa Maria, CA). Bacteria were harvested at mid-log phase and adjusted to 1×10^7^ cells/ml in Hank's Balanced Salt Solution (HBSS, Invitrogen). Kanamycin sulfate was added to all media at a final concentration of 200 µg/ml, where appropriate. Human monocytic cell line U937 (ATCC CRL-1593.2) was used for all assays and was grown in RPMI-1640 (GIBCO Laboratories) supplemented with heat-inactivated 10% fetal bovine serum (FBS) and 2 mM L-glutamine. Cells were seeded at 80% confluence into 75 cm^2^ tissue culture flasks, in 96-well plates or chamber glass slides, as needed. U937 macrophage monolayers were treated with 500 ng/ml phorbol 12-myristate 13-acetate (PMA, Sigma, St. Louis, MO) for 8 h to promote maturation and adherence and then monolayers were replenished with a new medium. After additional 24 h incubation, cells were infected with *M. tuberculosis* H37Rv or transposon mutants at MOI of 10. Infected and uninfected macrophages were collected at different time points for cytotoxicity assay, caspase activity or microarray assays. The percentage of phagocytized bacterium (wild-type or mutants) was calculated from controls grown under identical culture conditions for 3 h and 24 h in 24-well plates.

### *M. tuberculosis* transposon library construction, screening and sequencing

The *M. tuberculosis* transposon library was generated using the plasmid pTNGJC containing a temperature-sensitive origin of replication, as previously described [20]. Approximately 5,000 clones were stored in the library, in 50% glycerol at −80°C, and used for screening of clones for an increase of macrophage apoptosis compared to wild-type bacterium. U937 cells were treated with Phorbol 12-Myristate 13-Acetate (PMA, Sigma) and seeded at 1×10^5^ mononuclear cells/well in 96-well flat-bottomed tissue culture plates. Monolayers were infected with mutants and wild-type at a ratio of 10 bacteria∶1 cell and incubated for 2 h at 37°C and 5% CO_2_. The wells were washed three times with HBSS to remove extracellular bacteria and incubated at 37°C. After 5 days of infection, individual wells were visually screened for detached macrophages compare with control. Selected wells were processed for ELISA to confirm changes during *M. tuberculosis* mutant infection. *M. tuberculosis* genes interrupted by the Tn5367 transposon were identified using nonspecific nested suppression PCR method, previously reported by R. Tamme [21], and sequenced at the Central Service laboratories (CSL), Center for Gene Research and Biotechnology (CGRB), Oregon State University, Corvallis. Database search of the sequenced fragments was performed at the National Center for Biotechnology Information (www.ncbi.nih.gov), using BLAST network service.

### Complementation of *M. tuberculosis* 31G12 mutant

To complement the defect of 31G12 mutant, the *M. tuberculosis Rv3659c* gene, *Rv3656c–Rv3659c* and *Rv3654c–Rv3659c* genes were amplified from the wild-type H37Rv using GC-Rich PCR system (Roche). The PCR-generated fragments (Table 1) were cloned into *EcoR*I and *Hind*III sites of *Escherichia coli*-*Mycobacterium* shuttle vector pMV261+AprII [22], encoding apramycin resistance. The resulting plasmids pLD31G12-1, -2 and -3 were propagated in DH10B *E. coli* and then electroporated into *M. tuberculosis* 31G12 mutant. Transformants were selected on Middlebrook 7H10 agar plates containing 200 µg/ml of apramycin and screened by PCR using apramycin upper (GCATCGCATTCTTCGCATCC) and lower (GGCCCACTTGGACTGATCGA) primers for the 650 bp fragment from the apramycin-resistant gene. Constructed 31G12 clones were tested for restoration of the apoptosis phenotype using TUNEL assay.

**Table 1:** Sense (F) and Antisense (R) primers.

Experiment	Target	Primers	PCR product (bp)
Complementation:			
	Rv3659c	5′ tttgaattcatgctcggcgacaccgaa 3′ F	1059
		5′ cccaagctttcatgccgatgcccggct 3′ R	
	Rv3656c–Rv3659c	5′ tttgaattcatgctcggcgacaccgaa 3′ F	2700
		5′ cccaagcttctaaaccttggtgctgag 3′ R	
	Rv3654c–Rv3659c	5′ tttgaattcatgctcggcgacaccgaa 3′ F	3334
		5′ cccaagctttcaacccggtgtcgtggg 3′R	
Real-time PCR:			
	Rv3654c	5′ cttcgttagccgctgccg 3′ F	200
		5′ gggcaccttggccggccc 3′ R	
	Rv3656c	5′ atgttggtgatcaccatg 3′ F	200
		5′ ttggtgctgagcgcgcga 3′ R	
	Rv3659c	5′ gtgttgcgtcccgcgact 3′ F	200
		5′- tcgacgcacacgatccgc 3′ R	
Transcriptional analysis:			
	Rv3654c–Rv3657c	5′ tggccgtccaggttcggc 3′ F	960
		5′ gtcgaggtccaccaccct 3′ R	
	Rv3657c–Rv3659c	5′ gcggaccgtgggatgagt 3′ F	1000
		5′ acggccagcacgtcaagg 3′ R	
	Rv3659c–Rv3660c	5′ atgtgcgggcatgtgcgg 3′ F	800
		5′ ccgccgcagccaacgcgg 3′ R	

### Cytotoxicity assays

Cell Death Detection ELISA^PLUS^ and TUNEL assays (Roche) were used for detection of macrophage apoptosis. Necrosis, determined as lactate dehydrogenase (LDH) release from necrotic cells, was measured using the CytoTox 96 non-radioactive cytotoxicity assay (Promega), as previously described [10].

### Real-time PCR procedures and transcriptional analysis for *M. tuberculosis* genes

U937 macrophages were exposed to *M. tuberculosis* H37Rv for 30 min or infected for 3 h with MOI 100 bacteria∶1 cell. Both bacteria exposed to 7H9 broth and RPMI medium, for either 30 min or 3 h, were used as controls. Bacterial RNAs from control and experimental (exposed and intracellular) samples were extracted and processed for Real-time PCR, as previously described [23]. To determine whether the Rv3654c–Rv3660c locus produced a transcript containing seven genes, we arbitrarily chose approximately 1000 bp size intergenic regions of Rv3654c–Rv3657c, Rv3657c–Rv3659c and Rv3659c–Rv3660c and performed RT-PCR using intracellular bacterial RNA (Table S1).

### Caspase-8 activity

FLICE/Caspase-8 fluorimetric protease assay (Chemicon, Temecula, CA) was used to determine the changes in caspase-8 activity, 72 h following both *M. tuberculosis* wild-type and mutant infection of human macrophages. Treatment of U937 cells with human recombinant TNF-α protein (10^3^ U/ml) was used as a positive control. The assay was performed directly into 96-well tissue culture plate. Briefly, cells were resuspended in 50 µl of chilled lysis buffer, incubated on ice for 10 min, and 50 µl of 2× reaction butter containing 10 mM DTT was added. Each sample was mixed with 5 µl of 1 mM IETD-AFC (7-amino-4-trifluoromethyl coumarin) substrate to a final concentration of 50 µM and incubated at 37°C for 2 h. Results were measured using a Cytofluorimeter II (Biosearch, Bedford, MA) with 400 nm excitation and 505 nm emission filters.

CHeMICON's CaspaTag™ Caspase-8 *In Situ* Assay (Chemicon) was used for detection of active caspase-8 in U937 macrophages after transfection with *M. tuberculosis* proteins fused with Red Fluorescent Protein (RFP). 30× FLICA reagent solution at 1∶30 dilution in culture medium was added to transfected or non-transfected macrophage monolayers that were treated with human recombinant TNF-α protein (10^3^ U/ml) for 1 h at 37°C under 5% CO_2_. Hoechst stain 0.5% v/v (Chemicon) was added to each well for 5 min at 37°C, for nuclei labeling. Cells were washed three times with washing buffer, fixed in 1∶10 diluted fixative and analyzed under fluorescence microscope using band pass FITC filter for green fluorescence and UV-filter for apoptotic nuclei labeling with Hoechst stain.

### Detection of mitochondrial permeability

MitoLight™ Apoptosis assay (Chemicon) was used to detect the mitochondrial membrane disruption in *M. tuberculosis*-infected or -uninfected macrophages, according to the manufacturer's protocol. In some uninfected wells, staurosporine (Sigma) was added to trigger apoptosis (positive control). The lipophilic cationic dye was used to stain viable macrophage mitochondria (an assay dependent on the membrane potential) and was observed under fluorescence microscopy using Texas Red and FITC channels.

### Immunostaining of cytochrome C

The U937 cells were cultured in duplicate, using eight-chamber slides (NUNC). Macrophages infected with *M. tuberculosis* wild-type or with the mutant 31G12 for 72 h were washed three times with HBSS and fixed in 4% paraformaldehyde for 1 h. Cells were permeabilized with 1% Triton X-100 phosphate-buffered saline (PBS) for 5 min and blocked for 1 h in 2% bovine serum albumin. This was followed by a 5 h incubation with mouse monoclonal antibody against cytochrome C (1∶500, PharMingen, San Diego, CA). Macrophages were then washed and incubated with Texas Red-labeled goat anti-mouse IgG (1∶1000, Molecular Probe, Eugene, OR) for 1 h.

### Transfection experiments

Rv3654c (aa 84), Rv3655c (aa 125), Rv3656c (aa 68), Rv3657c (aa 576) and Rv3659c (aa 352) were subcloned into the EcoRI/BamHI sites of the pDsRed1-C1 vector and fused with Red Fluorescent Protein (CLONTECH). Resulting vectors pLDRv3654c, pLDRv3655c, pLDRv3656c, pLDRv3657c, pLDRv3659c, or the empty pDsRed1-C1 were transformed into U937 macrophages with Lipofectamine 2000 transfection reagents using the manufacturer's protocol (Invitrogen).

### Western blot analysis of *M. tuberculosis*-secreted proteins

The pJAM2:Rv3654c and pJAM2:Rv3655c plasmid constructs were introduced into *M. tuberculosis* and kanamycin-resistant colonies grown in M63 medium (7.6×10^−2^ M (NH_4_)_2_SO_4_, 0.5 M KH_2_PO_4_, 5.8×10^−6^ M FeSO_4_.7H_2_O, pH 7) supplemented with 1 mM MgSO_4_, 0.5% Tween-80 and 2% succinate for non-induced cultures or 2% succinate and 2% acetamide for induced cultures. Bacteria were grown for 21 days, after which cells were harvested, washed two times and used for infection of U937 macrophages.

Macrophages were infected with *M. tuberculosis* pJAM2:Rv3654c, or pJAM2:Rv3655c, induced and non-induced clones for 48 h. Cell cultures were lysed with 0.05% SDS and centrifuged at 3,000× *g* for 15 min to remove bacterial pellet from the suspension. Pre-cleared cell lysates were incubated with His.tag primary agarose conjugate antibodies, overnight at 4°C. Samples were resolved by electrophoresis on 12.5% SDS-PAGE gels, transferred to nitrocellulose membranes and blocked overnight with 5% blocking reagent. The secondary mouse horseradish peroxidase-linked whole (IgG) antibody was used at a dilution 1∶5000. Reactivity was assessed with Hyperfilm ECL detection reagents (Amersham) followed by autoradiography.

### His-tagged pull-down assay

Macrophage lysates were prepared and labeled as previously described [22], mixed with 1 mg of purified His.tag-Rv3654c or His.tag-Rv3655c proteins and 60 µl of His.tag agarose conjugate rabbit polyclonal IgG beads (500 g/ml with 25% AG). The samples were incubated at 4°C overnight, spun down, washed three times with PBS and processed for electrophoresis. The pulled-down proteins were subjected for In-Gel Tryptic Digestion (PIERCE, Rockford, IL) and sequencing by electrospray ionization mass spectrometry (ESI-MS/MS).

### Mass Spectrometric sequencing

Proteins were analyzed at Environmental Health Science Center (EHS) Mass Spectrometry Facility, Oregon State University, Corvallis, by chromatography and electrospray ionization mass spectrometry (ESI-MS/MS). Tandem MS was performed with ESI, and mass spectra were acquired by using quadrupole–time-of-flight (Q-TOF) Global Ultima system from Micromass (Manchester, UK), operated with a spray voltage of 3.5 kV. Digested protein samples were mixed 1∶1 (v/v) with 0.1% formic acid, 0.005% trifluoroacetic acid, and 3% acetonitrile in H_2_O (solvent A). A Symmetry 300 C 18 trap from Dionex (Sunnyvale, CA) and 75-m inner-diameter PicoFrit column from New Objective (Woburn, MA), packed in-house with Jupiter C 5 from Phenomenex (Torrance, CA), were used for the ESI experiments. The LC program consisted of a gradient from 3% to 35% B over 60 min, to 70% B at 65 min, and finally 95% B from 70 to 80 min. Solvent B contained 0.1% formic acid and 0.005% trifluoroacetic acid in 80% acetonitrile. Data-dependent MS/MS was generated using a 0.5-sec MS survey scan and 2.5-sec MS/MS scans on the three most abundant peaks found in the survey scan. A database search was performed using Mascot (Matrix Science, London, UK) and IPI human (Bioinformatics Solutions, Inc., PEAKS studio, Canada) software.

### PSF and ALK proteins

The anti-polypyrimidine tract-binding protein (PTB)-associated splicing factor (PSF) antibody (Santa Cruz Biotechnologies, Inc.) and FITC-conjugated mouse anti-human NPM-ALK/ALK (Anaplastic Lymphoma Kinase) antibody (ALK1, BD Pharmingen) were used for fluorescence microscopy studies. Briefly, 4×10^5^ cells were seeded on eight-chamber slides and infected or not with rhodamine-labeled wild-type or 31G12 bacteria. One well was transfected with either pLDRv3654c or pLDRv3655c plasmid to investigate the expression of either PSF or ALK proteins, respectively. After 48 h of infection, cells were fixed for 1 h in 4% formaldehyde, permeabilized with 0.1% Triton X-100 for 10 min and blocked in 2% bovine serum albumin, followed by a 2 h incubation with rabbit monoclonal antibody against PSF (1∶500) or FITC-conjugated monoclonal ALK1 antibody (1∶500). Fluorescein-labeled goat anti-rabbit IgG (1∶2000, Molecular Probe, Eugene, OR) was used as secondary antibody for PSF protein. Results were confirmed by Western bolt analysis of immunoprecipitated samples (experimental and control) for PSF protein.

### Inactivation of PSF by siRNA

To examine the role of PSF in caspase-8 activation, transfection of macrophages with PSF siRNA (Santa Cruz) was performed using Santa Cruz Biotechnology reagents, in accordance with the manufacturer's recommendations. Isolated 1×10^6^/ml macrophages were seeded in six-well tissue culture plates, washed once with siRNA transfection medium and replaced with transfection medium containing PSF siRNA transfection reagent mixture. Untreated and control siRNA (scrabbled sequences)-transfected cells were used as negative controls. Cells were cultured in the absence or presence of recombinant TNF-α protein. PSF and caspase-8 protein expressions were determined by Western blot analysis.

### Macrophage assays

The U937 cells were made adherent to plates by incubating them with 500 ng/ml phorbol 12-myristate 13-acetate. U937 cells (5×10^5^) were infected with *M. tuberculosis* H37Rv or mutant 31G12 (MOI of 10), and extracellular bacteria were removed by washing after 1 h. Intracellular bacteria were allowed to grow up to 5 days. Several wells were lysed at 1 h and plated onto 7H10 agar to quantify the intact inoculums. At 2 and 5 days, both attached and detached macrophages were collected, lysed and CFU counts were recorded.

### Statistical analysis

All experiments were carried out in duplicate and repeated at least two times. Mean values and standard deviations were calculated to describe the data population. Significance of the differences between experimental groups and control groups was analyzed by the Student's *t*-test or ANOVA. P values of <0.05 were considered significant.

## Results

### Mutant library screening and analysis

The *M. tuberculosis* transposon bank was partially screened (5,000 mutants) for the lack of ability to inhibit macrophage apoptosis. The initial selection was performed based on the observation that infected U937 phagocytes would become apoptotic after 4–5 days and detach from the tissue culture wells. TUNEL and ELISA assays confirmed that 99% of detached cells were, in fact, undergoing apoptosis. We visually identified 54 clones in which the number of detached cells in the tissue culture wells differed significantly from monolayers infected with the wild-type bacterium. The selected clones were further evaluated by TUNEL and ELISA assays. Thirty-two of the 54 clones lacked the ability to inhibit macrophage apoptosis. Previously published studies have shown that *M. tuberculosis* inhibits apoptosis similarly in U937, THP-1 and human monocyte-derived macrophages [10].

**Figure 1:**
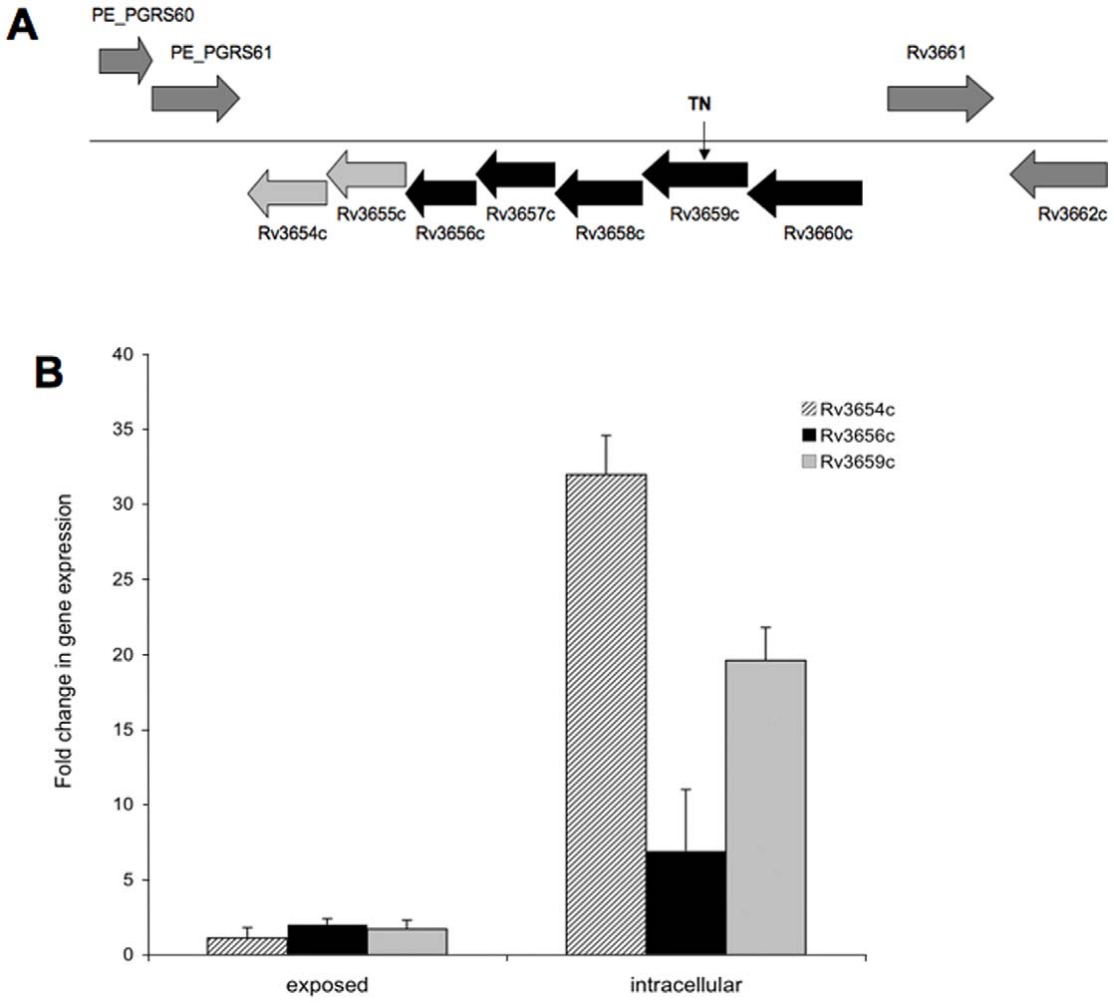
Transposon-inactivated region in the bacterial chromosome. **A** Gene organization of 31G12 mutant associated with induction of macrophage apoptosis. The operon contains seven genes, including four genes (*Rv3657c–Rv3660c*) that resemble a pilus assembly proteins, type II/IV secretion system proteins, as well as tight adherence genes of TadZ, TadA-C, respectively; Rv3656c transmembrane protein and Rv3654c, Rv3655c genes that encode secreted proteins. The arrow indicates the gene disrupted by the insertion of Tn5367 transposon (TN). **B** *M. tuberculosis* gene expression following 30 min exposure to macrophages or 3 h infection. Total RNAs from broth-grown bacteria, as well as from intracellular- and macrophage-exposed bacteria, were used to determine the copy numbers of cDNAs for target and reference genes. Data were analyzed on Ct values basis for each sample and normalized with an internal housekeeping gene control, 16S rRNA.

Identified mutants are shown in Table 2. The sequencing of 31G12 mutant demonstrated that the *Rv3659c* gene was inactivated. The gene belongs to an operon of seven genes (Figure 1A). The operon is part of the same transcriptional unit, as evidenced by RT-PCR. In this operon the genes *Rv3657c* to *Rv3660c* have significant homology to many bacterial type IV pili and to *Actinobacillus actinomycetemcomitans* pilus assembly *tad* genes. The inactivated *Rv3659c* is part of the pili assembly genes, and has homology to *VirB11*. The downstream genes in the operon are a putative transmembrane protein (Rv3656c) and two secreted proteins (Rv3654 and Rv3655c). The *Rv3654c–Rv3659c* genes are significantly induced in the intracellular environment, compared with the gene expression in bacteria exposed to RPMI-1640 supplemented by 10% FBS (Figure 1B).

**Table 2:** Transposon-based list of *M. tuberculosis* genes related to increased apoptosis during infection of macrophages.

Clone	*M. tuberculosis* H_37_R_v_ Gene	Product description
Lipid metabolism		
2F6	Rv0129c or *fbpC*	Secreted antigen 85-C FBPC
9D6	Rv0166 or *fadD5*	Probable fatty-acid-CoA ligase fadD5. Involved in lipid degradation.
27G1/30H10	Rv0468 or *fadB2*	3-Hydroxybutyryl-CoA dehydrogenase. Butyrate/Butanol-producing pathway
4G2	Rv2246 or *kasB*	3-oxoacyl-[Acyl-carrier protein] synthase 2. Involved in fatty acid biosynthesis.
24F10	Rv3801c or *fadD32*	Probable fatty-acid-CoA synthetase
29D1	Rv3825c or *pks2*	Polyketide synthase
Information pathways		
4E3	Rv0041 or *leuS*	Leucyl-tRNA synthetase. Involved in translation mechanism.
2B9	Rv0058 or *dnaB*	Replicative DNA helicase. Participates in initiation and elongation during chromosome replication.
Cell wall and cell processes		
9B9	Rv1476	Possible membrane protein
8B6	Rv2690c	Conserved integral membrane alanine, valine and leucine rich protein
30G12	Rv2729c	Conserved integral membrane alanine, valine and leucine rich protein
22C12/ 23D1	Rv3823c or *mmpL8*	Probable conserved integral membrane transport protein
Intermediary metabolism and respiration		
41H7	Rv1285 or *cysD*	Sulfate adenylyltransferase subunit 2. Involved in sulfate activation pathway.
40D11	Rv1286 or *cysN*	Bifunctional enzyme: Sulfate adenyltransferase (subunit 1)
3E4	Rv1304 or *atpB*	ATP synthase a chain ATPB. Key component of the proton channel.
24D1	Rv1436 or *gap*	Probable Glyceraldehyde 3-phosphate dehydrogenase
40H7	Rv1437 or *pgk*	Phosphoglycerate kinase
41H4	Rv1880c or *cyp140*	Probable cytochrome p450 140 cyp140. Contains cytochrome p450 cysteine heme-iron ligand signature.
43A2/45F4	Rv2454c	Putative oxidoreductase (beta subunit).
8H10	Rv3518c or *cyp142*	Probable cytochrome p450 142 cyp142. Contains cytochrome p450 cysteine heme-iron ligand signature.
50F4	Rv3545c or *cyp125*	Probable cytochrome p450 125 cyp125. They oxidize a variety of structurally unrelated compounds, including steroids, fatty acids, and xenobiotics.
4G3	Rv3859c or *gltB*	Probable involved in glutamate biosynthesis.
Conserved hypotheticals		
18E11	Rv2567	Conserved hypothetical alanine and leucine rich protein.
23G6/30G7	Rv2751	Conserved hypothetical protein
2G2	Rv3354	Conserved hypothetical protein.
20A11	Rv3365c	Conserved hypothetical protein. Contains neutral zinc metallopeptidases, zinc-binding region signature.
31G12/39H7	Rv3659c	Conserved hypothetical protein

### Effects of 31G12 complementation

In order to identify whether the observed phenotype was due to the inactivation of *Rv3659c* or other downstream genes, we created three constructs covering genes encoding for the structural, transmembrane and secreted proteins in the region. Macrophages were infected with complemented 31G12 clones, and monitored for five days for apoptotic changes by the TUNEL assay. In parallel experiments, apoptosis was induced in wild-type infected and uninfected control cells by TNF-α (Table 3). While *M. tuberculosis* H37Rv infected macrophages triggered a 24% greater level of apoptosis than uninfected macrophages after five days, infection with the 31G12 mutant led to an increase of apoptosis, 66% compared with uninfected controls. The 31G12 mutant, complemented with pLD31G12-3, a clone containing all six downstream genes of the operon, was able to inhibit apoptosis in a similar fashion to *M. tuberculosis* H37Rv. In contrast, complementation of 31G12 with pLD31G12-1 or pLD31G12-2 plasmid did not revert the phenotype (Table 3). In addition, we observed that *M. tuberculosis* H37Rv infection of macrophages was associated with suppression of TNF-α-induced macrophage apoptosis as shown on the Table 3.

**Table 3:** *M. tuberculosis* infection inhibits TNF-α-induced apoptosis in macrophages.

Infections and Treatment	% Apoptosis/200 cells (TUNEL assay)
	U937 (after 5 days)
No bacteria	12.5±5
Treated with TNF-α	82±1^a^
Infected with wild-type	36.5±2^b^
Infected with 31G12	78.5±2^c^
Infected with complement pLD31G12-1 (Rv3659c)	75±4^c^
Infected with complement pLD31G12-2 (Rv3656c–Rv3659c)	80±2^c^
Infected with complement pLD31G12-3 (Rv3654c–Rv3659c)	49±3^d^
Infected with wild-type and treatment with TNF-α	40±1^d^

Macrophage monolayers were treated with 10^3^ U/ml (1 ng/ml) of human recombinant TNF-α.

a p<0.05 compared to apoptosis with no bacteria.

b p<0.05 compared to macrophage treated with TNF-α.

c p<0.05 compared to monolayer infected with wild-type H37Rv.

d p<0.05 compared to monolayer infected with the strain 31G12 or treated with TNF-α.

### Changes in the extrinsic and intrinsic pathways of apoptosis

**Figure 2:**
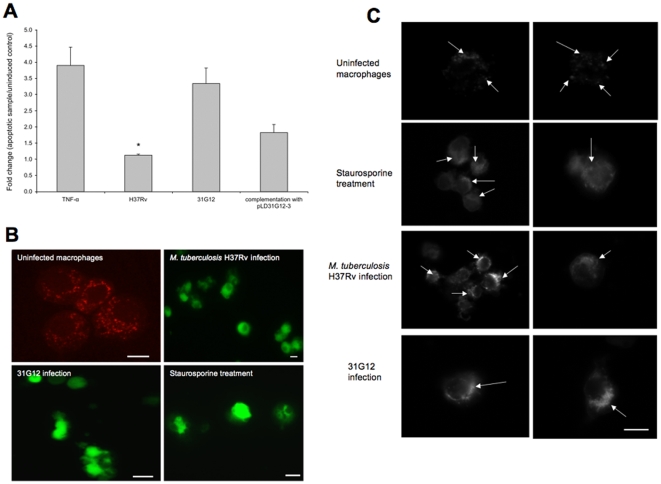
Effect on apoptotic pathways. **A** *M. tuberculosis* infection prevents caspase-8 activation in macrophages. U937 cells were infected or uninfected and/or treated with recombinant TNF-α protein, and fold change of caspase-8 activation was calculated according the manufacturer's protocol. The *, statistically significant difference between TNF-α-treated and *M. tuberculosis*-infection groups (p<0.05). **B** Immunofluorescence analysis of mitochondrial transmembrane disruption after *M. tuberculosis* H37Rv, 31G12 infection and staurosporine treatment, compared with uninfected macrophages. Red dots indicate undamaged mitochondria in uninfected cells. Bar, 10 µm. **C** *M. tuberculosis* infection promotes cytochrome C release from the mitochondria into the cytoplasm of U937 cells. Macrophages were infected with wild-type bacterium or 31G12 mutant and were then stained for cytochrome C. Staurosporine treatment was used as positive control. The arrows on the punctuated staining indicate cytochrome C localization in the mitochondria in uninfected macrophages; diffuse staining shows characteristic mitochondrial cytochrome C release in *M. tuberculosis* H37Rv- or 31G12-infected cells, similar to the staurosporine treatment. Two images were included for each experimental group. Bar, 10 µm.

It was observed that, while recombinant TNF-α-treated and 31G12-infected macrophages showed caspase-8 activation, significant suppression of caspase-8 activation was observed in both macrophages infected with wild-type bacterium and with the complemented 31G12 strain containing the pLD31G12-3 construct (Figure 2A).

Using mitochondrial permeability and cytochrome C assays (Figure 2, B and C) as indicators of the activation of the intrinsic pathway, we observed that there was no differential effect between the wild-type and the knockout bacterium on macrophage apoptosis. Figure 2B shows that the lipophilic cation dye is accumulated in the mitochondria of uninfected cells (bright red fluorescence). The mitochondrial membrane was damaged in *M. tuberculosis* H37Rv- and 31G12-infected macrophages, where the dye had diffused in the cytoplasm giving bright green fluorescence.

Immunolabeling of cytochrome C in uninfected macrophages showed a granular pattern; whereas, a diffuse cytoplasmic staining was observed in macrophages infected with *M. tuberculosis* wild-type or 31G12 mutant. The pattern was similar to the control cells treated with staurosporine (Figure 2C).

### Resistance to TNF-α-mediated apoptosis during *M. tuberculosis* infection

**Figure 3:**
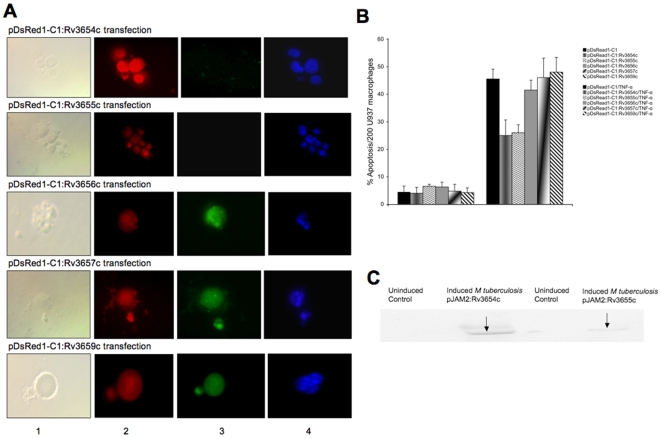
Macrophage transfection affects apoptosis. **A** CaspaTag™ *In Situ* analysis of U937 macrophages for caspase-8 activation and nuclear changes during transfection with *M. tuberculosis* proteins. While transfection with *M. tuberculosis Rv3654c* and *Rv3655c* genes during TNF-α treatment of macrophages suppressed caspase-8 activation and nuclear fragmentation, *M. tuberculosis Rv3656c*-, *Rv3657c*- and *Rv3659c*- transfected and TNF-α-treated macrophages showed caspase-8 activation and nuclear morphological changes typical of apoptosis. Colors in the columns: (1) Phase contrast, (2) Red – transfection, (3) Green – caspase-8 activation and (4) Blue – nuclear staining with Hoechst stain. Bar, 10 µm. **B** Effect of *M. tuberculosis*-secreted proteins on caspase-8 activation during macrophage apoptosis. U937 cells were transfected with *Rv3654c–Rv3659c* genes for 8 h and then treated with human recombinant TNF-α protein (0.5 µM). Active caspase-8 was quantified using *In Situ* Assay kit (Chemicon). **C** Analysis of secreted proteins in the cytoplasm of infected macrophages. U937 cells were infected with *M. tuberculosis* containing pJAM2:Rv3654c or pJAM2:Rv3655c over-expressed vectors at an MOI 1∶10. Concentrated bacterial and cell lysate-free supernatants were subjected to Western blotting using His-tag antibody.

To investigate whether the putatively secreted proteins in the operon were capable of inhibiting macrophage apoptosis by the extrinsic pathway, U937 cells were transfected with pDsRed1-C1 plasmid, containing *Rv3654c*, *Rv3655c*, *Rv3656c*, *Rv3657c* or *Rv3659c* genes, and evaluated for caspase-8 activation and nuclear fragmentation following treatment with human recombinant TNF-α. *M. tuberculosis* putatively secreted the proteins (Rv3654c, Rv3655c) significantly suppressed TNF-α-induced apoptosis (Figure 3A), which could not be observed in cells transfected with *Rv3657c and Rv3659c*. Components of type IV pili apparatus, or the transmembrane gene-encoding protein *Rv3656c*. The findings were confirmed by quantification of apoptotic cells (Figure 3B).

### Secretion of Rv3654c and Rv3655c

To investigate whether Rv3654c and Rv3655c were secreted into the macrophage cytoplasm, we fused both proteins with 6-His-tag and over-expressed them in *M. tuberculosis* using pJAM2 vector. Infected macrophages were lysed by day 2, separated from the intracellular bacteria and processed for Western blot analysis. Rv3654c and Rv3655c proteins were detected in the macrophage cytoplasmic fraction (Figure 3C).

### Host proteins interacting with *M. tuberculosis* secreted proteins

**Figure 4:**
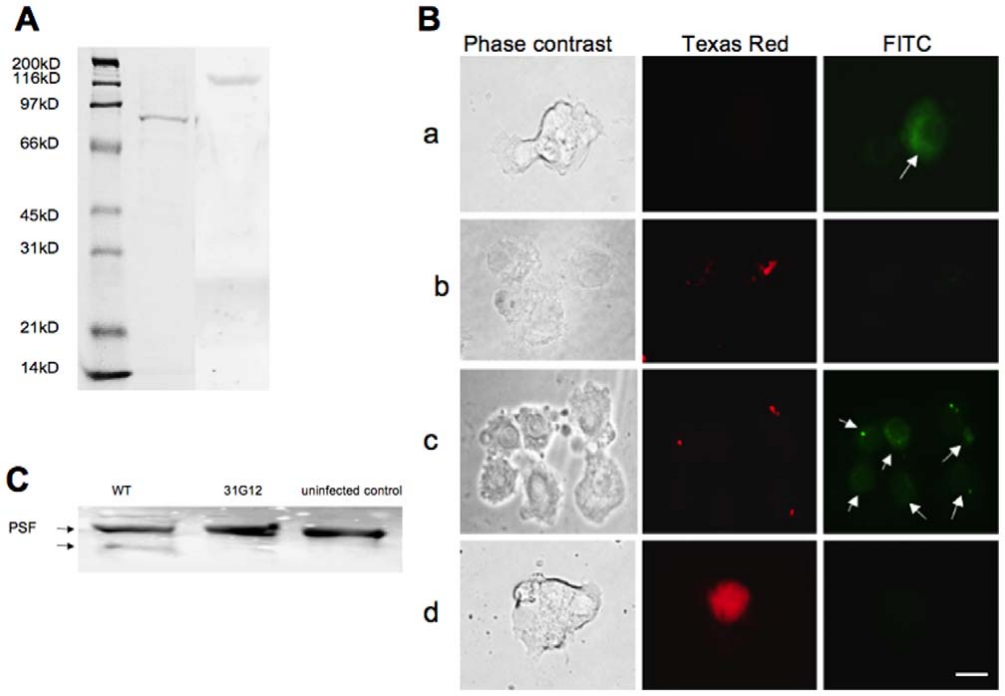
His-tagged pull-down analysis. **A** His-tagged pull-down assay of cellular proteins interacting with *M. tuberculosis* Rv3654c and Rv3655c proteins identified 76kDa PSF (1) and 175kDa ALO17 (2), respectively. **B** PSF protein levels in U937 cells. Macrophages were uninfected (a) or infected with *M. tuberculosis* wild-type (b); 31G12 mutant (c); and transfected with pLDRv3654c (d). Bacteria (red) were labeled with rhodamine and the PSF protein was visualized by fluorescein conjugated secondary antibody (green). Bar, 10 µm. **C** Western blot analysis of PSF protein after 24 h of infection with *M. tuberculosis* wild-type and 31G12 mutant.

His-tagged pull-down assay (Figure 4A) and mass spectrometric analysis (Table 4) of host cell proteins interacting with *M. tuberculosis* Rv3654c and Rv3655c identified 76kDa PSF (splicing factor, proline- and glutamine-rich; Polypyrimidine tract-binding protein-associated-splicing factor) and 175kDa ALO17 (ALK lymphoma oligomerization partner on chromosome 17), respectively.

**Table 4:** Amino acid sequences of macrophage proteins interacting with *M. tuberculosis* Rv3654c and Rv3655c.

Protein (Accession number)	Sequence
76kDa Splicing factor, proline- and glutamine-rich; Polypyrimidine tract-binding protein-associated-splicing factor (PSF); UniProt P23246	FGQGGAGPVGGQGPRGMGP
175kDa ALK lymphoma oligomerization partner on chromosome 17 (ALO17); UniProt Q9HCF4	QFPAEHGWKESLLGDMEWRLTK

Using immunofluorescence, macrophages infected with *M. tuberculosis* H37Rv, 31G12 mutant or macrophages transfected with pLDRv3654c showed differences regarding PSF protein expression levels (Figure 4B). PSF protein was expressed at a high level and diffused in the cytoplasm of uninfected cells (Figure 4B, a), while within macrophages containing the pLDRv3654c and also in macrophages infected with H37Rv (Figure 4B, d and b), significantly reduced levels of PSF protein at 48 h time point was observed. In contrast, the level of PSF protein is elevated in 31G12 mutant-infected cells (Figure 4B, c). Further analysis by Western blot showed that the wild-type bacterium cleaves PSF protein, which was not seen in phagocytic cells infected with the 31G12 mutant (Figure 4C).

**Figure 5:**
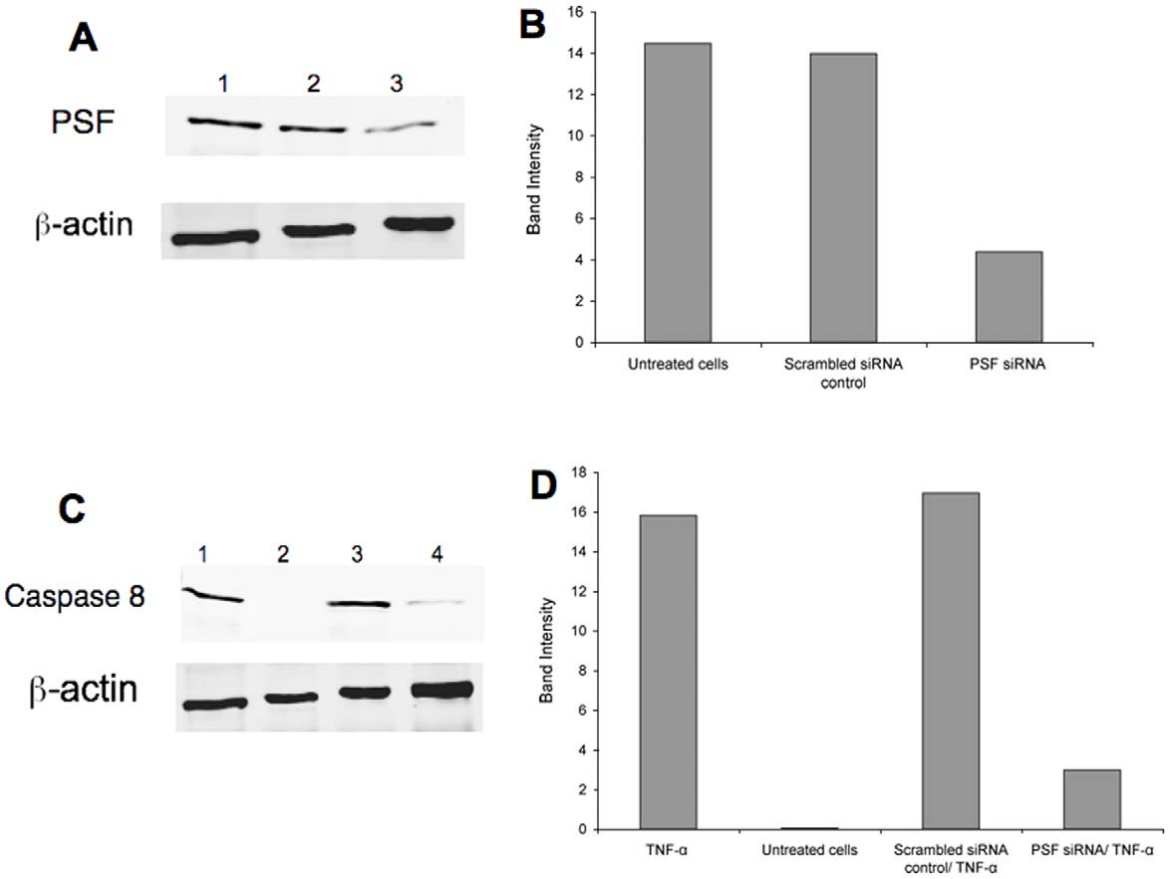
Macrophage transfection with siRNA. (**A**) PSF protein expression levels by Western blot analysis: 1. Untreated cells, 2. Scrambled siRNA control transfection, 3. PSF siRNA transfection. **B** PSF protein band intensity levels measured with Li-Cor Odyssey Imaging software. **C** Caspase-8 protein expression levels by Western blot analysis: 1. Recombinant TNF-α treatment (positive control), 2. Untreated cells, 3. Scrambled siRNA control transfection and recombinant TNF-α treatment, 4. PSF siRNA transfection and recombinant TNF-α treatment. **D** Caspase-8 protein band intensity levels measured with Li-Cor Odyssey Imaging software.

Using the interference siRNA system, we then inactivated PSF to identify its role in caspase-8 activation. As observed in Figures 5A and 5B, the expression of PSF was significantly reduced in macrophages transfected with siRNA. Figures 5C and 5D show that inactivation of PSF leads to a significant reduction of caspase-8 protein in macrophages.

ALO17, a protein of unknown function, has recently been described as a novel fusion partner of ALK [24]. We used fluorescent labeling of ALK to examine whether it formed fusion with ALO17. ALK was not expressed in *M. tuberculosis*-infected macrophages, as determined by fluorescence microscopy and RT-PCR using primers designed for the fused transcripts, as previously described [25]. Real-time PCR was also performed for the expression of ALO17. During *M. tuberculosis* wild-type infection of macrophages, ALO17 gene showed 5.3-fold higher expression over the uninfected control.

### Survival in macrophages

To verify the differences in macrophage survival during 31G12 and the wild-type bacterium infection, intracellular bacteria were cultured from detached (apoptotic) and attached macrophages at two different time points. The results (Table 5) show that, while the wild-type bacterium grows within attached macrophages, the 31G12 mutant loses viability over time in apoptotic macrophages, demonstrating attenuated phenotype.

**Table 5:** Mutants with impaired ability to inhibit apoptosis have an attenuated phenotype in macrophages.

		CFU/10^5^ macrophages	
Bacterial Strain	Macrophage	2 days	5 days
H37Rv	adherent	2.6±0.5×10^5^	3.1±0.4×10^6^
	detached	NA	2.8±0.5×10^5^ b
31G12	adherent	1.9±0.5×10^5^	2.8±0.6×10^6^
	detached	1.2±0.5×10^5^	2.6±0.2×10^2^ a

a p<0.01 compared with the number of bacteria in attached macrophages.

b p<0.01 compared with the number of intracellular 31G12 mutant at day 5.

NA: Not Available. The number of detached macrophages was small and accurate quantification was not possible.

### Intrinsic pathway of apoptosis and macrophage necrosis

Since *M. tuberculosis* is known to exit macrophages by inducing cell necrosis, we decided to examine whether pharmacological inhibition of macrophage apoptosis by the intrinsic pathway could have any impact on the phagocytic cell necrosis induced by *M. tuberculosis*.

**Figure 6:**
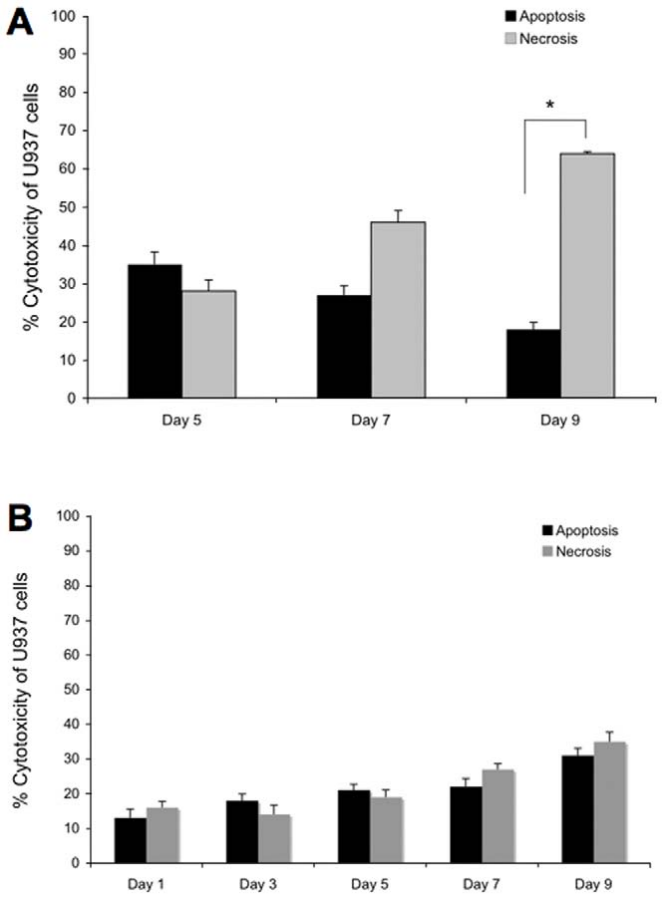
Macrophage apoptosis and necrosis. **A** Comparison of the levels of macrophage apoptosis and necrosis during *M. tuberculosis* H37Rv infection at later time points. *M. tuberculosis* induced significantly more necrosis of macrophages (64±0.5%) at day 9 (p<0.05) than apoptosis (18±1.8%), compared with uninfected controls. The decreased level of apoptosis during the infection with virulent *M. tuberculosis* is related to duration of macrophage infection; however, *M. tuberculosis* triggers a greater level of necrosis at later time points than apoptosis. **B** Inhibitory effect of Z-LEHD-FMK.TFA (caspase-9 inhibitor) on necrosis levels of macrophages. Comparisons of *M. tuberculosis* H37Rv-induced apoptosis and necrosis at different time points did not show significant induction of necrosis after treatment of infected macrophages with caspase-9 inhibitor.

In agreement with previously published data, *M. tuberculosis* H37Rv infection was associated with reduced macrophage necrosis after 1 and 3 days of infection compared with the level of apoptosis at the same time points (data not shown); however, *M. tuberculosis* triggered a greater level of necrosis than apoptosis at later time points (Figure 6A). To identify weather *M. tuberculosis*-induced necrosis of macrophages is related to the intrinsic apoptotic pathway, host cells were treated with irreversible caspase-9 inhibitor Z-LEHD-FMK.TFA (Sigma) and levels of cytotoxicity were determined by 3, 5, 7 and 9 days post-infection. *M. tuberculosis* H37Rv-infected macrophages did not undergo necrosis after blocking the intrinsic pathway with the caspase-9 inhibitor (Figure 6B). The data indicate that inhibition of the intrinsic pathway of apoptosis at later time points had direct correlation to increased levels of necrosis during *M. tuberculosis* infection of U937 cells.

## Discussion

*M. tuberculosis* is a pathogen that evolved several different strategies to survive within host cells. Among them are the ability to impair nitric oxide delivery to the phagosome [5], the prevention of phagosome-lysosome fusion [2], [3], and the capacity to interfere with autophagy [7]. Macrophage apoptosis is a host mechanism of innate immunity that has been shown to eliminate *M. tuberculosis* [13]. A number of studies, however, have suggested that *M. tuberculosis* infection of macrophages either induces or blocks apoptosis [10], [14], which apparently correlates with strain virulence and microbial burden and certainly reflects the different models used. We have evaluated apoptosis and necrosis caused by *M. tuberculosis* H37Rv in two cell lines (U937 and THP-1) and in primary human monocyte-derived macrophages [10]. In our hands, *M. tuberculosis* infection of the three macrophages above was associated with inhibition of apoptosis by the extrinsic pathway but did not interfere in a significant manner with the intrinsic pathway. In the current work, by screening a transposon mutant library, we identified two *M. tuberculosis* proteins (Rv3654c, Rv3655c) that are exported outside of the bacterial phagosome by a putative secretion apparatus. The choice of these mutants, from among all others, was due to the interesting nature of the chromosomal region containing the inactivated Rv3659c gene. Of note, the “type IV pili” operon that contains Rv3654c and Rv3655c is only upregulated when the bacterium is within macrophages. The type II/IV secretion system identified in *M. tuberculosis* is well conserved in many microorganisms, and in some cases, it has been associated with bacterial attachment to epithelial cells [26]. In the majority of the bacteria, however, the function of this chromosomal region is currently unknown. Our findings suggest a novel function for the region that contains genes encoding for four secretion apparatus structural proteins, one transmembrane protein, and two secreted proteins, which interfere with macrophage apoptosis.

The secreted proteins Rv3654c and Rv3655c interact with host PSF and ALO17, respectively, and as consequence, interfere with extrinsic pathway of apoptosis, as demonstrated by transfecting macrophages with the bacterial proteins. PSF is a factor essential for pre-mRNA splicing [24]. PSF has been shown to regulate apoptosis by influencing caspase activity at the translational level. During cell apoptosis, PSF remains intact and phosphorylated, which is not expected of a splicing factor, suggesting a possible secondary function. In fact, we were able to demonstrate that inactivation of PSF leads to significant decrease of caspase-8 expression. Our study also shows that the wild-type bacterium cleaves PSF after 48 h of infection, in contrast with the 31G12 mutant, making it the probable mechanism of inactivation. In fact, the work by Shav-Tal and colleagues suggests that PSF is sensitive to proteolysis and can be degraded [27]. Alternative explanation for the decrease of PSF would be related to the phosphorylation of PSF, which occurs following macrophage apoptosis. It could make at least some of the protein undetected by antibodies.

Rv3655c recognizes AL017, a protein usually associated with ALK (anaplastic lymphoma kinase) [25]. Various fusion partners of ALK and AL017 have been described, all of them with anti-apoptotic function of cells [28]. Once ALK is activated in the cytoplasm, it migrates into the nucleus and phosphorylates NIPA (nuclear interacting partner of ALK) protein. NIPA protein has been found to inhibit PI3-kinase/AKT anti-apoptotic signaling pathway and then inhibit caspases. Although we could not show the ALK-AL017 interaction, we demonstrated that the ALO17 gene is highly expressed in macrophages, after infection with *M. tuberculosis* H37Rv.

Apoptosis is now considered as one of the host mechanisms of innate immunity. Intracellular pathogens, which overcome many of the killing strategies of phagocytic cells, such as reactive oxygen and reactive nitrogen products, antimicrobial peptides and autophagy, had to evolve strategies to survive the apoptosis of the host cell. Because host cells have many triggering mechanisms for apoptosis, and at least three apoptotic pathways are currently known, we can assume that *M. tuberculosis* had to adapt to all in order to remain viable within macrophages. By screening a transposon mutant library, we identified several deficient mutants that very likely have different or complementary mechanisms to suppress apoptosis. The overlapping functions of bacterial genes point to the importance of the strategy, which, in addition, influence the outcome of infection. Our results also unveil a plausible important strategy of the pathogen. By inhibiting the translation phase of the extrinsic pathway of apoptosis, other proteins co-expressed with caspases may remain intact to perform potentially significant functions, some of them still unknown. In addition, the level of inhibition is the most efficacious one, not depending on gene regulation, which can be complex. The fact that *M. tuberculosis* does not seem to significantly suppress apoptosis by the intrinsic pathway is one of the reasons for much of the diverse results in the field. In fact, if one does not use mutants to understand the effect on the pathway, conflicting results are very likely. Our findings suggest that *M. tuberculosis* acts in different ways to manipulate apoptosis in macrophages. Suppression and, possibly, stimulation of apoptotic pathways by the pathogen depend on the phase of the host cell infection and have important roles in the outcome of the condition, as demonstrated in this study and elsewhere [29]. This complex interaction can also be responsible for some of the discrepancies among the published results. Data from *in vivo* observation supports that clinical strains capable of inhibiting apoptosis are more virulent [29]. The finding that *M. tuberculosis* uses the intrinsic pathway of apoptosis as a strategy to induce macrophage necrosis explains, at least in part, the fact that the pathogen appears to inhibit apoptosis selectively through the extrinsic pathway. It is interesting that the bacterium uses the activation of the intrinsic pathway during a very defined phase of infection to induce necrosis. One can propose that there is some advantage to following this particular strategy, although we currently cannot understand it. We do know that the proteins ESAT-6 and CFP-10 appear to participate in the process of macrophage necrosis [30], [31]. *M. tuberculosis* is now accepted as a pathogen that escapes the macrophage during a phase of the intracellular life, probably infecting other cells. All the evidence thus far supports the idea that the pathogen triggers necrosis or some lesion of the macrophage surface [32]. The genes linked to this process and the mechanisms are still under investigation. Other pathogens, such as *Legionella pneumophila*, inhibit macrophage apoptosis by targeting pro-death members of the Bcl-2 protein family [33]. In this case, SidF, a *Legionella* protein, binds to Bcl-rambo and BNIP-3, interfering with the intrinsic apoptotic pathway.

Our model agrees with the model described by Chen and colleagues [13] that the suppressive effect of *M. tuberculosis* infections on apoptosis occurs in the extrinsic but not in the intrinsic pathway. We also found that *M. tuberculosis* uses the intrinsic pathway to trigger necrosis. Future investigation will attempt to enhance our understanding of the complex interaction of *M. tuberculosis* with different apoptotic pathways.

## Supporting Information

Table S1 (0.04 MB DOC)
