# Supplementary material for: Secreted Mycobacterium tuberculosis Rv3654c and Rv3655c Proteins Participate in the Suppression of Macrophage Apoptosis
Source: PLoS One. 2010 May 4;5(5):e10474. doi: 10.1371/journal.pone.0010474 (PMC2864267; doi:10.1371/journal.pone.0010474)
Supplement: Table S1 — (0.04 MB DOC) [file pone.0010474.s001.doc]

Table. Sense and antisense primers.

Experiment Target Primers PCR product (bp)

Complementation:

Rv3654c-Rv3659c 5’- tttgaattcatgctcggcgacaccgaa -3’ 3334

5’- cccaagctttcaacccggtgtcgtggg -3’

Real-time PCR:

Rv3654c 5’- cttcgttagccgctgccg -3’ 200

5’- gggcaccttggccggccc -3’

Rv3656c 5’- atgttggtgatcaccatg -3’ 200

5’- ttggtgctgagcgcgcga -3’

Rv3659c 5’- gtgttgcgtcccgcgact -3’ 200

5’- tcgacgcacacgatccgc -3’

Transcriptional analysis:

Rv3654c-Rv3657c 5’- tggccgtccaggttcggc -3’ 960

5’- gtcgaggtccaccaccct -3’

Rv3657c-Rv3659c 5’- gcggaccgtgggatgagt -3’ 1000

5’- acggccagcacgtcaagg -3’

Rv3659c-Rv3660c 5’- atgtgcgggcatgtgcgg -3’ 800

5’- ccgccgcagccaacgcgg -3’
